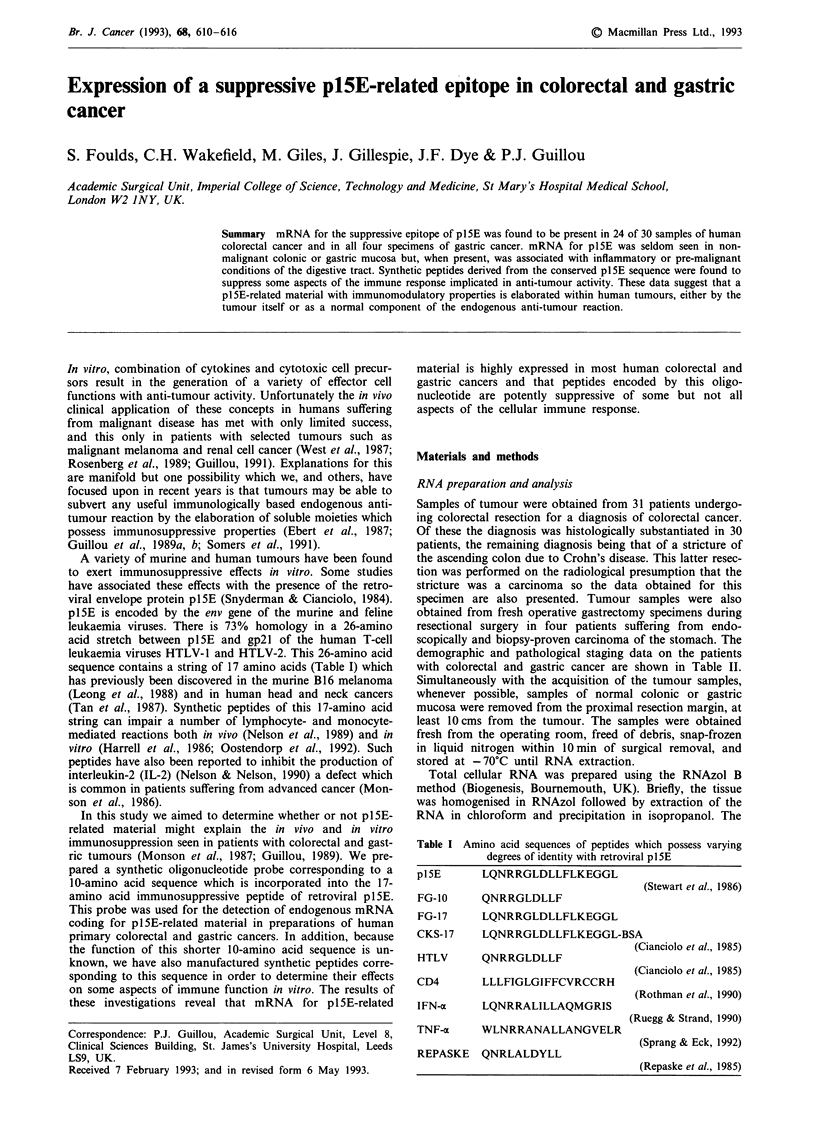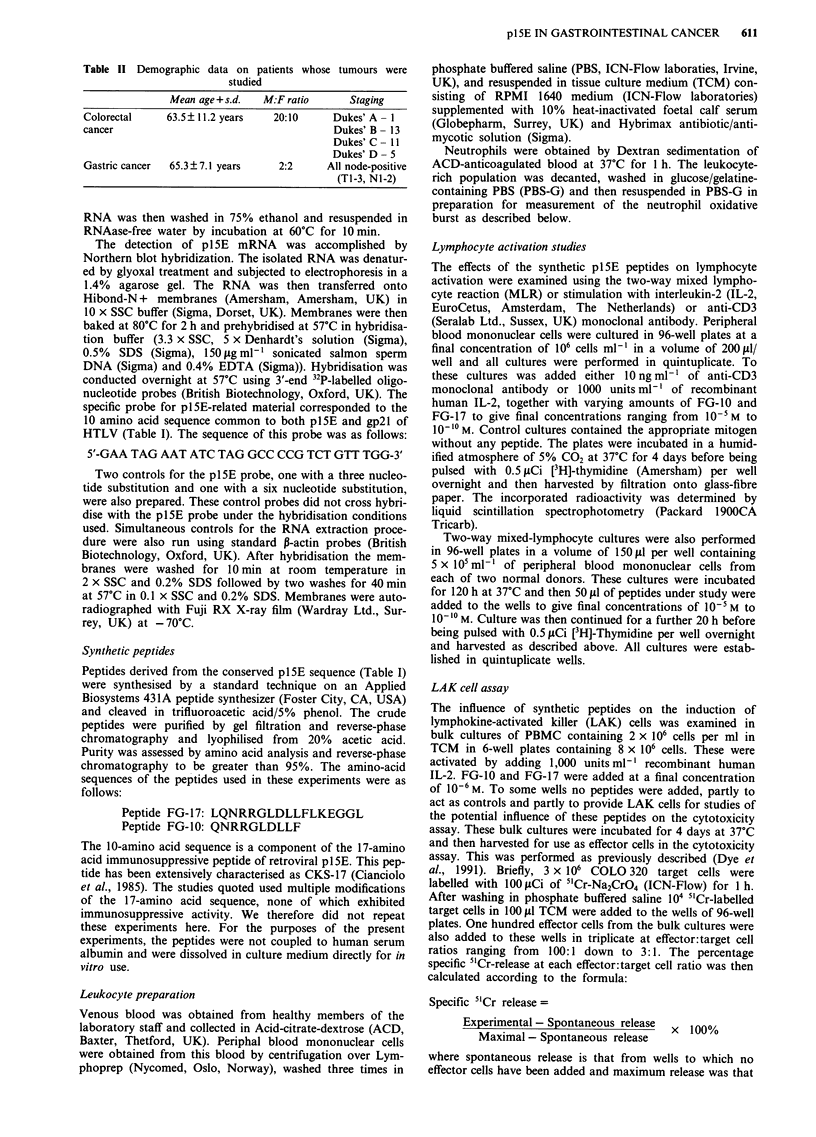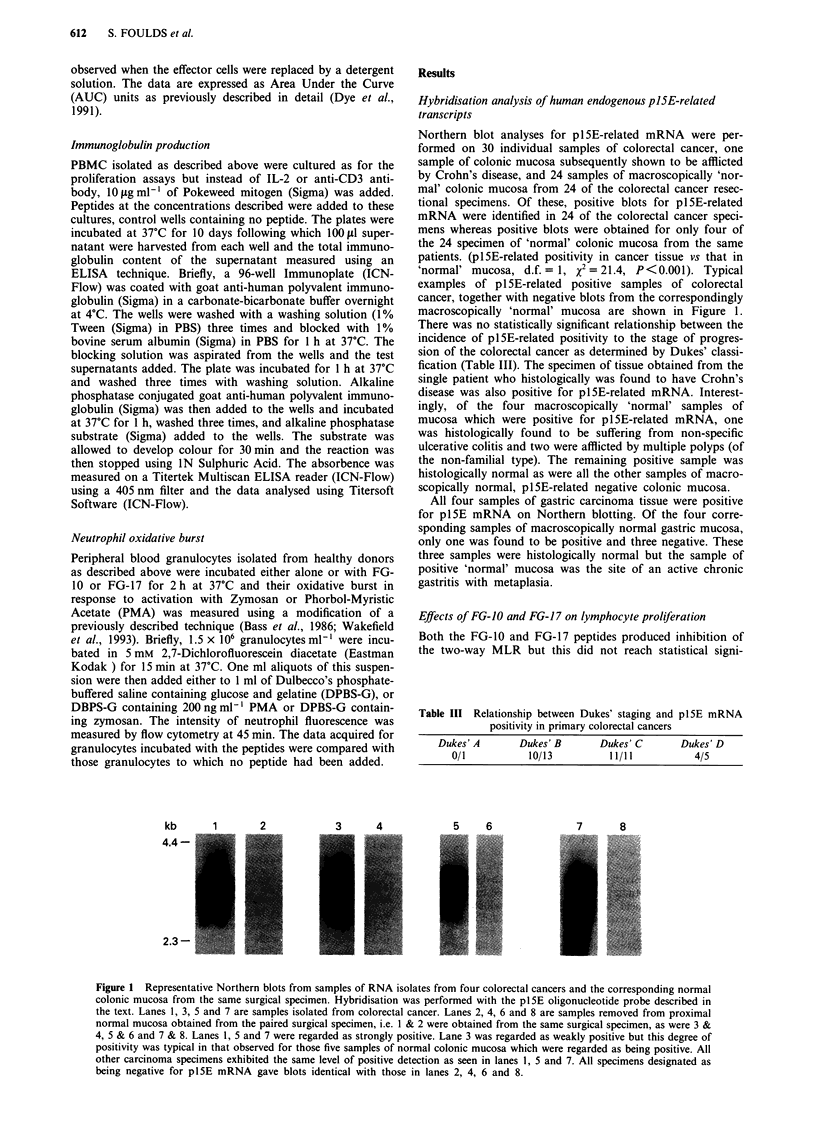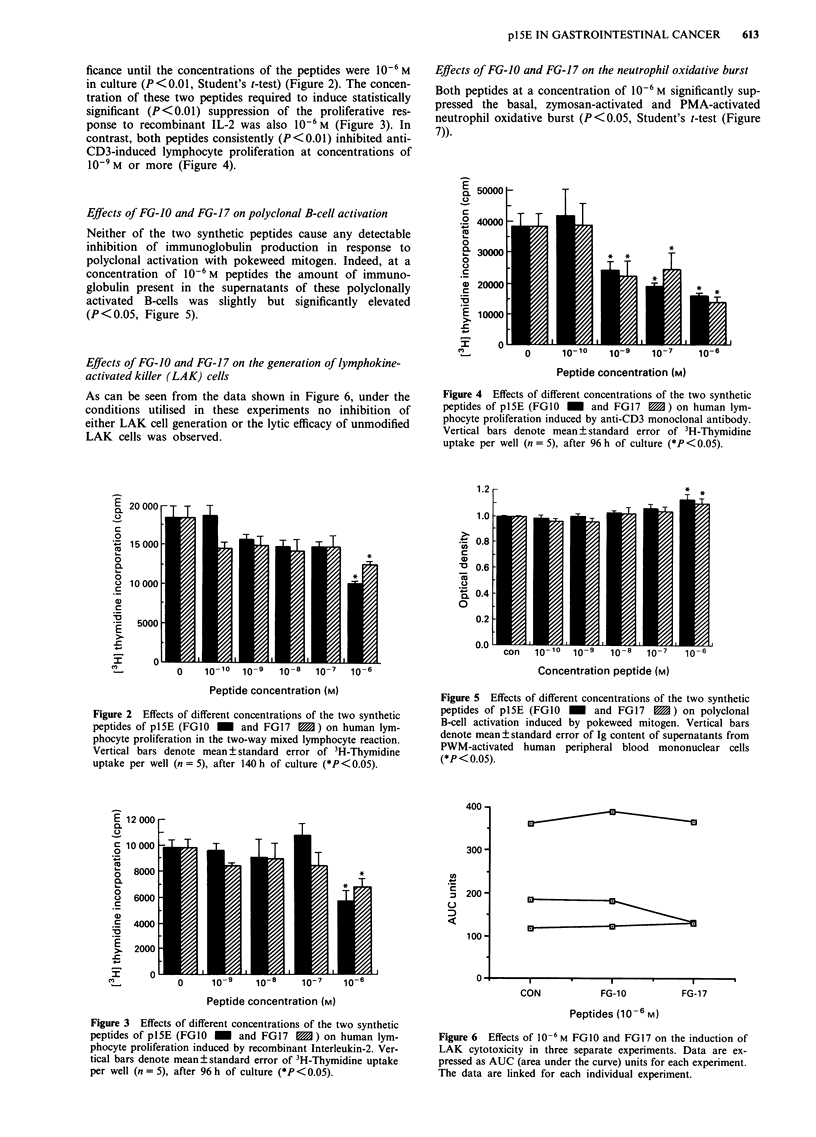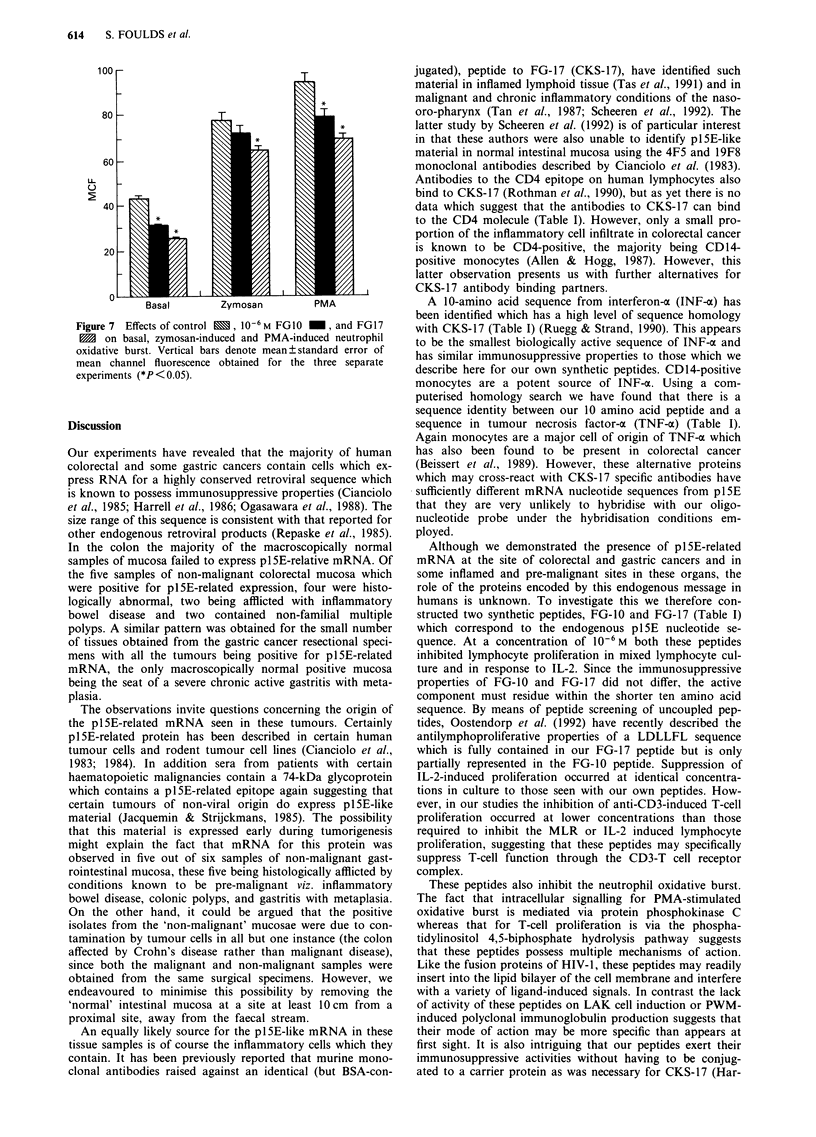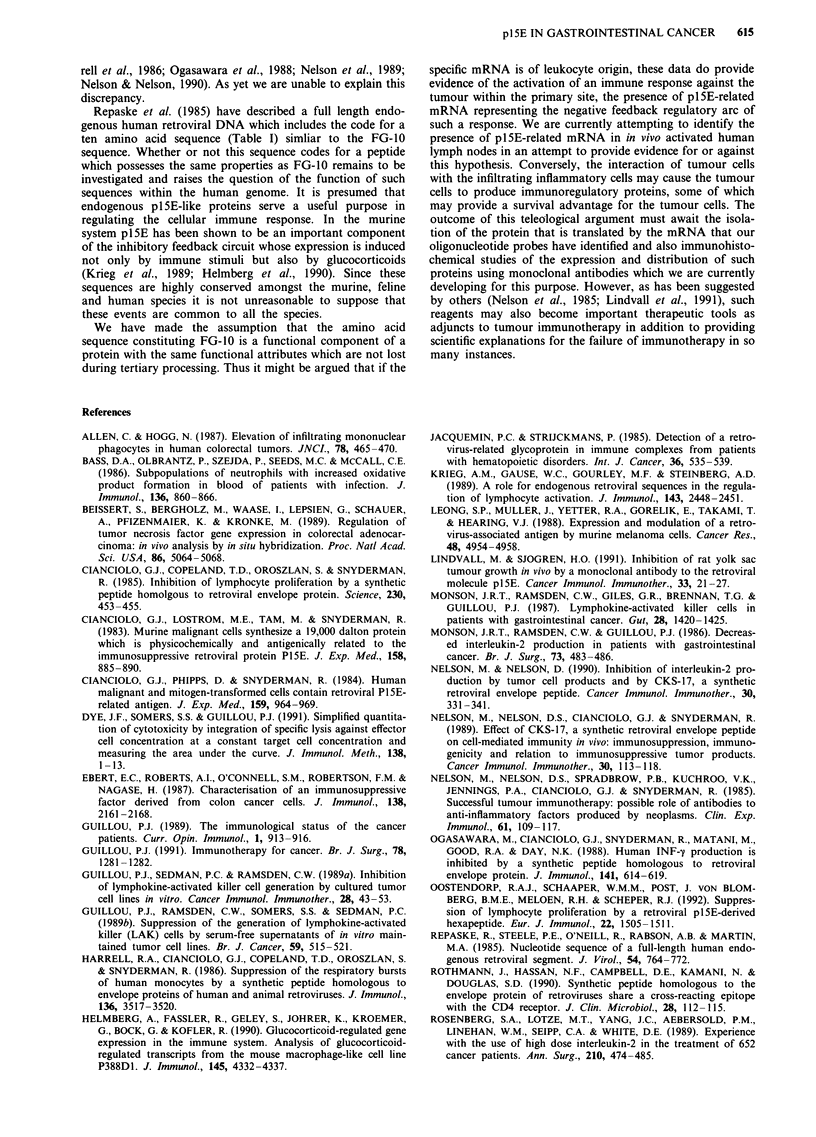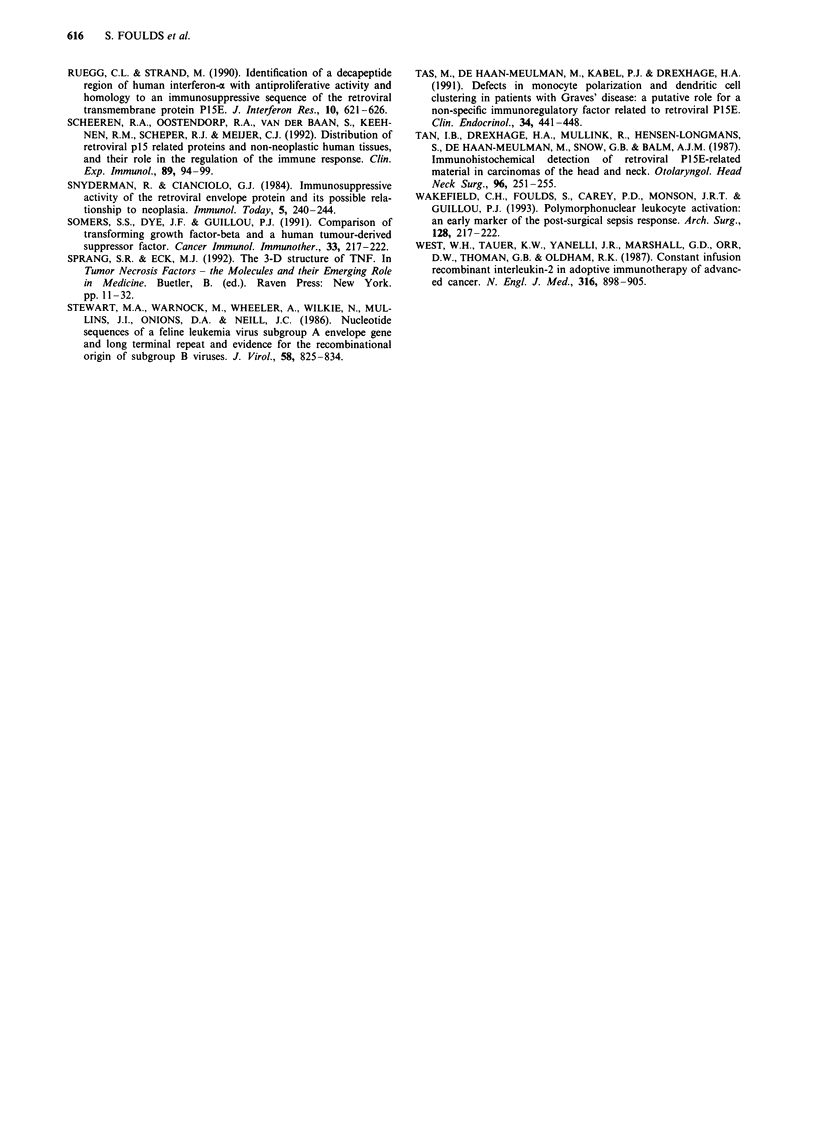# Expression of a suppressive p15E-related epitope in colorectal and gastric cancer.

**DOI:** 10.1038/bjc.1993.395

**Published:** 1993-09

**Authors:** S. Foulds, C. H. Wakefield, M. Giles, J. Gillespie, J. F. Dye, P. J. Guillou

**Affiliations:** Academic Surgical Unit, Imperial College of Science, Technology and Medicine, St Mary's Hospital Medical School, London, UK.

## Abstract

**Images:**


Br. J. Cancer (1993), 68, 610-616 © Macmillan Press Ltd., 1993

### Expression of a suppressive p15E-related epitope in colorectal and gastric cancer

S. Foulds, C.H. Wakefield, M. Giles, J. Gillespie, J.F. Dye & P.J. Guillou

*Academic Surgical Unit, Imperial College of Science, Technology and Medicine, St Mary's Hospital Medical School, London W2 1NY, UK.*

**Summary** mRNA for the suppressive epitope of p15E was found to be present in 24 of 30 samples of human colorectal cancer and in all four specimens of gastric cancer. mRNA for p15E was seldom seen in non-malignant colonic or gastric mucosa but, when present, was associated with inflammatory or pre-malignant conditions of the digestive tract. Synthetic peptides derived from the conserved p15E sequence were found to suppress some aspects of the immune response implicated in anti-tumour activity. These data suggest that a p15E-related material with immunomodulatory properties is elaborated within human tumours, either by the tumour itself or as a normal component of the endogenous anti-tumour reaction.

*In vitro*, combination of cytokines and cytotoxic cell precursors result in the generation of a variety of effector cell functions with anti-tumour activity. Unfortunately the *in vivo* clinical application of these concepts in humans suffering from malignant disease has met with only limited success, and this only in patients with selected tumours such as malignant melanoma and renal cell cancer (West *et al.*, 1987; Rosenberg *et al.*, 1989; Guillou, 1991). Explanations for this are manifold but one possibility which we, and others, have focused upon in recent years is that tumours may be able to subvert any useful immunologically based endogenous anti-tumour reaction by the elaboration of soluble moieties which possess immunosuppressive properties (Ebert *et al.*, 1987; Guillou *et al.*, 1989*a*, *b*; Somers *et al.*, 1991).

A variety of murine and human tumours have been found to exert immunosuppressive effects *in vitro*. Some studies have associated these effects with the presence of the retroviral envelope protein p15E (Snyderman & Cianciolo, 1984). p15E is encoded by the *env* gene of the murine and feline leukaemia viruses. There is 73% homology in a 26-amino acid stretch between p15E and gp21 of the human T-cell leukaemia viruses HTLV-1 and HTLV-2. This 26-amino acid sequence contains a string of 17 amino acids (Table I) which has previously been discovered in the murine B16 melanoma (Leong *et al.*, 1988) and in human head and neck cancers (Tan *et al.*, 1987). Synthetic peptides of this 17-amino acid string can impair a number of lymphocyte- and monocyte-mediated reactions both *in vivo* (Nelson *et al.*, 1989) and *in vitro* (Harrell *et al.*, 1986; Oostendorp *et al.*, 1992). Such peptides have also been reported to inhibit the production of interleukin-2 (IL-2) (Nelson & Nelson, 1990) a defect which is common in patients suffering from advanced cancer (Monson *et al.*, 1986).

In this study we aimed to determine whether or not p15E-related material might explain the *in vivo* and *in vitro* immunosuppression seen in patients with colorectal and gastric tumours (Monson *et al.*, 1987; Guillou, 1989). We prepared a synthetic oligonucleotide probe corresponding to a 10-amino acid sequence which is incorporated into the 17-amino acid immunosuppressive peptide of retroviral p15E. This probe was used for the detection of endogenous mRNA coding for p15E-related material in preparations of human primary colorectal and gastric cancers. In addition, because the function of this shorter 10-amino acid sequence is unknown, we have also manufactured synthetic peptides corresponding to this sequence in order to determine their effects on some aspects of immune function *in vitro*. The results of these investigations reveal that mRNA for p15E-related material is highly expressed in most human colorectal and gastric cancers and that peptides encoded by this oligonucleotide are potently suppressive of some but not all aspects of the cellular immune response.

Correspondence: P.J. Guillou, Academic Surgical Unit, Level 8, Clinical Sciences Building, St. James's University Hospital, Leeds LS9, UK.
Received 7 February 1993; and in revised form 6 May 1993.

### Materials and methods

### *RNA preparation and analysis*

Samples of tumour were obtained from 31 patients undergoing colorectal resection for a diagnosis of colorectal cancer. Of these the diagnosis was histologically substantiated in 30 patients, the remaining diagnosis being that of a stricture of the ascending colon due to Crohn's disease. This latter resection was performed on the radiological presumption that the stricture was a carcinoma so the data obtained for this specimen are also presented. Tumour samples were also obtained from fresh operative gastrectomy specimens during resectional surgery in four patients suffering from endoscopically and biopsy-proven carcinoma of the stomach. The demographic and pathological staging data on the patients with colorectal and gastric cancer are shown in Table II. Simultaneously with the acquisition of the tumour samples, whenever possible, samples of normal colonic or gastric mucosa were removed from the proximal resection margin, at least 10 cms from the tumour. The samples were obtained fresh from the operating room, freed of debris, snap-frozen in liquid nitrogen within 10 min of surgical removal, and stored at −70°C until RNA extraction.

Total cellular RNA was prepared using the RNAzol B method (Biogenesis, Bournemouth, UK). Briefly, the tissue was homogenised in RNAzol followed by extraction of the RNA in chloroform and precipitation in isopropanol. The

**Table I** Amino acid sequences of peptides which possess varying degrees of identity with retroviral p15E

| | | |
|---|---|---|
| p15E | LQNRRGLDLLFLKEGGL | (Stewart *et al.*, 1986) |
| FG-10 | QNRRGLDLLF | |
| FG-17 | LQNRRGLDLLFLKEGGL | |
| CKS-17 | LQNRRGLDLLFLKEGGL-BSA | (Cianciolo *et al.*, 1985) |
| HTLV | QNRRGLDLLF | (Cianciolo *et al.*, 1985) |
| CD4 | LLLFIGLGIFFCVRCCRH | (Rothman *et al.*, 1990) |
| IFN-α | LQNRRALILLAQMGRIS | (Ruegg & Strand, 1990) |
| TNF-α | WLNRRANALLANGVELR | (Sprang & Eck, 1992) |
| REPASKE | QNRLALDYLL | (Repaske *et al.*, 1985) |

**Table II** Demographic data on patients whose tumours were studied

| | *Mean age+s.d.* | *M:F ratio* | *Staging* |
|---|---|---|---|
| Colorectal cancer | 63.5±11.2 years | 20:10 | Dukes' A - 1<br>Dukes' B - 13<br>Dukes' C - 11<br>Dukes' D - 5 |
| Gastric cancer | 65.3±7.1 years | 2:2 | All node-positive (T1-3, N1-2) |

RNA was then washed in 75% ethanol and resuspended in RNAase-free water by incubation at 60°C for 10 min.

The detection of p15E mRNA was accomplished by Northern blot hybridization. The isolated RNA was denatured by glyoxal treatment and subjected to electrophoresis in a 1.4% agarose gel. The RNA was then transferred onto Hibond-N+ membranes (Amersham, Amersham, UK) in 10 × SSC buffer (Sigma, Dorset, UK). Membranes were then baked at 80°C for 2 h and prehybridised at 57°C in hybridisation buffer (3.3 × SSC, 5 × Denhardt's solution (Sigma), 0.5% SDS (Sigma), 150 μg ml$^{-1}$ sonicated salmon sperm DNA (Sigma) and 0.4% EDTA (Sigma)). Hybridisation was conducted overnight at 57°C using 3′-end $^{32}$P-labelled oligonucleotide probes (British Biotechnology, Oxford, UK). The specific probe for p15E-related material corresponded to the 10 amino acid sequence common to both p15E and gp21 of HTLV (Table I). The sequence of this probe was as follows:

5′-GAA TAG AAT ATC TAG GCC CCG TCT GTT TGG-3′

Two controls for the p15E probe, one with a three nucleotide substitution and one with a six nucleotide substitution, were also prepared. These control probes did not cross hybridise with the p15E probe under the hybridisation conditions used. Simultaneous controls for the RNA extraction procedure were also run using standard β-actin probes (British Biotechnology, Oxford, UK). After hybridisation the membranes were washed for 10 min at room temperature in 2 × SSC and 0.2% SDS followed by two washes for 40 min at 57°C in 0.1 × SSC and 0.2% SDS. Membranes were autoradiographed with Fuji RX X-ray film (Wardray Ltd., Surrey, UK) at −70°C.

*Synthetic peptides*

Peptides derived from the conserved p15E sequence (Table I) were synthesised by a standard technique on an Applied Biosystems 431A peptide synthesizer (Foster City, CA, USA) and cleaved in trifluoroacetic acid/5% phenol. The crude peptides were purified by gel filtration and reverse-phase chromatography and lyophilised from 20% acetic acid. Purity was assessed by amino acid analysis and reverse-phase chromatography to be greater than 95%. The amino-acid sequences of the peptides used in these experiments were as follows:

Peptide FG-17: LQNRRGLDLLFLKEGGL
Peptide FG-10: QNRRGLDLLF

The 10-amino acid sequence is a component of the 17-amino acid immunosuppressive peptide of retroviral p15E. This peptide has been extensively characterised as CKS-17 (Cianciolo *et al.*, 1985). The studies quoted used multiple modifications of the 17-amino acid sequence, none of which exhibited immunosuppressive activity. We therefore did not repeat these experiments here. For the purposes of the present experiments, the peptides were not coupled to human serum albumin and were dissolved in culture medium directly for *in vitro* use.

*Leukocyte preparation*

Venous blood was obtained from healthy members of the laboratory staff and collected in Acid-citrate-dextrose (ACD, Baxter, Thetford, UK). Periphal blood mononuclear cells were obtained from this blood by centrifugation over Lymphoprep (Nycomed, Oslo, Norway), washed three times in phosphate buffered saline (PBS, ICN-Flow laboraties, Irvine, UK), and resuspended in tissue culture medium (TCM) consisting of RPMI 1640 medium (ICN-Flow laboratories) supplemented with 10% heat-inactivated foetal calf serum (Globepharm, Surrey, UK) and Hybrimax antibiotic/antimycotic solution (Sigma).

Neutrophils were obtained by Dextran sedimentation of ACD-anticoagulated blood at 37°C for 1 h. The leukocyte-rich population was decanted, washed in glucose/gelatine-containing PBS (PBS-G) and then resuspended in PBS-G in preparation for measurement of the neutrophil oxidative burst as described below.

*Lymphocyte activation studies*

The effects of the synthetic p15E peptides on lymphocyte activation were examined using the two-way mixed lymphocyte reaction (MLR) or stimulation with interleukin-2 (IL-2, EuroCetus, Amsterdam, The Netherlands) or anti-CD3 (Seralab Ltd., Sussex, UK) monoclonal antibody. Peripheral blood mononuclear cells were cultured in 96-well plates at a final concentration of $10^6$ cells ml$^{-1}$ in a volume of 200 μl/well and all cultures were performed in quintuplicate. To these cultures was added either 10 ng ml$^{-1}$ of anti-CD3 monoclonal antibody or 1000 units ml$^{-1}$ of recombinant human IL-2, together with varying amounts of FG-10 and FG-17 to give final concentrations ranging from $10^{-5}$ M to $10^{-10}$ M. Control cultures contained the appropriate mitogen without any peptide. The plates were incubated in a humidified atmosphere of 5% $CO_2$ at 37°C for 4 days before being pulsed with 0.5 μCi [$^3$H]-thymidine (Amersham) per well overnight and then harvested by filtration onto glass-fibre paper. The incorporated radioactivity was determined by liquid scintillation spectrophotometry (Packard 1900CA Tricarb).

Two-way mixed-lymphocyte cultures were also performed in 96-well plates in a volume of 150 μl per well containing $5 \times 10^5$ ml$^{-1}$ of peripheral blood mononuclear cells from each of two normal donors. These cultures were incubated for 120 h at 37°C and then 50 μl of peptides under study were added to the wells to give final concentrations of $10^{-5}$ M to $10^{-10}$ M. Culture was then continued for a further 20 h before being pulsed with 0.5 μCi [$^3$H]-Thymidine per well overnight and harvested as described above. All cultures were established in quintuplicate wells.

*LAK cell assay*

The influence of synthetic peptides on the induction of lymphokine-activated killer (LAK) cells was examined in bulk cultures of PBMC containing $2 \times 10^6$ cells per ml in TCM in 6-well plates containing $8 \times 10^6$ cells. These were activated by adding 1,000 units ml$^{-1}$ recombinant human IL-2. FG-10 and FG-17 were added at a final concentration of $10^{-6}$ M. To some wells no peptides were added, partly to act as controls and partly to provide LAK cells for studies of the potential influence of these peptides on the cytotoxicity assay. These bulk cultures were incubated for 4 days at 37°C and then harvested for use as effector cells in the cytotoxicity assay. This was performed as previously described (Dye *et al.*, 1991). Briefly, $3 \times 10^6$ COLO 320 target cells were labelled with 100 μCi of $^{51}$Cr-$Na_2CrO_4$ (ICN-Flow) for 1 h. After washing in phosphate buffered saline $10^4$ $^{51}$Cr-labelled target cells in 100 μl TCM were added to the wells of 96-well plates. One hundred effector cells from the bulk cultures were also added to these wells in triplicate at effector:target cell ratios ranging from 100:1 down to 3:1. The percentage specific $^{51}$Cr-release at each effector:target cell ratio was then calculated according to the formula:

$$\text{Specific } ^{51}\text{Cr release} = \frac{\text{Experimental} - \text{Spontaneous release}}{\text{Maximal} - \text{Spontaneous release}} \times 100\%$$

where spontaneous release is that from wells to which no effector cells have been added and maximum release was that observed when the effector cells were replaced by a detergent solution. The data are expressed as Area Under the Curve (AUC) units as previously described in detail (Dye et al., 1991).

*Immunoglobulin production*

PBMC isolated as described above were cultured as for the proliferation assays but instead of IL-2 or anti-CD3 antibody, $10\,\mu g\,ml^{-1}$ of Pokeweed mitogen (Sigma) was added. Peptides at the concentrations described were added to these cultures, control wells containing no peptide. The plates were incubated at 37°C for 10 days following which 100 µl supernatant were harvested from each well and the total immunoglobulin content of the supernatant measured using an ELISA technique. Briefly, a 96-well Immunoplate (ICN-Flow) was coated with goat anti-human polyvalent immunoglobulin (Sigma) in a carbonate-bicarbonate buffer overnight at 4°C. The wells were washed with a washing solution (1% Tween (Sigma) in PBS) three times and blocked with 1% bovine serum albumin (Sigma) in PBS for 1 h at 37°C. The blocking solution was aspirated from the wells and the test supernatants added. The plate was incubated for 1 h at 37°C and washed three times with washing solution. Alkaline phosphatase conjugated goat anti-human polyvalent immunoglobulin (Sigma) was then added to the wells and incubated at 37°C for 1 h, washed three times, and alkaline phosphatase substrate (Sigma) added to the wells. The substrate was allowed to develop colour for 30 min and the reaction was then stopped using 1N Sulphuric Acid. The absorbence was measured on a Titertek Multiscan ELISA reader (ICN-Flow) using a 405 nm filter and the data analysed using Titersoft Software (ICN-Flow).

*Neutrophil oxidative burst*

Peripheral blood granulocytes isolated from healthy donors as described above were incubated either alone or with FG-10 or FG-17 for 2 h at 37°C and their oxidative burst in response to activation with Zymosan or Phorbol-Myristic Acetate (PMA) was measured using a modification of a previously described technique (Bass et al., 1986; Wakefield et al., 1993). Briefly, $1.5 \times 10^6$ granulocytes $ml^{-1}$ were incubated in 5 mM 2,7-Dichlorofluorescein diacetate (Eastman Kodak ) for 15 min at 37°C. One ml aliquots of this suspension were then added either to 1 ml of Dulbecco's phosphate-buffered saline containing glucose and gelatine (DPBS-G), or DBPS-G containing $200\,ng\,ml^{-1}$ PMA or DPBS-G containing zymosan. The intensity of neutrophil fluorescence was measured by flow cytometry at 45 min. The data acquired for granulocytes incubated with the peptides were compared with those granulocytes to which no peptide had been added.

### Results

*Hybridisation analysis of human endogenous p15E-related transcripts*

Northern blot analyses for p15E-related mRNA were performed on 30 individual samples of colorectal cancer, one sample of colonic mucosa subsequently shown to be afflicted by Crohn's disease, and 24 samples of macroscopically 'normal' colonic mucosa from 24 of the colorectal cancer resectional specimens. Of these, positive blots for p15E-related mRNA were identified in 24 of the colorectal cancer specimens whereas positive blots were obtained for only four of the 24 specimen of 'normal' colonic mucosa from the same patients. (p15E-related positivity in cancer tissue vs that in 'normal' mucosa, d.f. = 1, $\chi^2 = 21.4$, $P < 0.001$). Typical examples of p15E-related positive samples of colorectal cancer, together with negative blots from the correspondingly macroscopically 'normal' mucosa are shown in Figure 1. There was no statistically significant relationship between the incidence of p15E-related positivity to the stage of progression of the colorectal cancer as determined by Dukes' classification (Table III). The specimen of tissue obtained from the single patient who histologically was found to have Crohn's disease was also positive for p15E-related mRNA. Interestingly, of the four macroscopically 'normal' samples of mucosa which were positive for p15E-related mRNA, one was histologically found to be suffering from non-specific ulcerative colitis and two were afflicted by multiple polyps (of the non-familial type). The remaining positive sample was histologically normal as were all the other samples of macroscopically normal, p15E-related negative colonic mucosa.

All four samples of gastric carcinoma tissue were positive for p15E mRNA on Northern blotting. Of the four corresponding samples of macroscopically normal gastric mucosa, only one was found to be positive and three negative. These three samples were histologically normal but the sample of positive 'normal' mucosa was the site of an active chronic gastritis with metaplasia.

*Effects of FG-10 and FG-17 on lymphocyte proliferation*

Both the FG-10 and FG-17 peptides produced inhibition of the two-way MLR but this did not reach statistical signi-

**Table III** Relationship between Dukes' staging and p15E mRNA positivity in primary colorectal cancers

| *Dukes' A* | *Dukes' B* | *Dukes' C* | *Dukes' D* |
|---|---|---|---|
| 0/1 | 10/13 | 11/11 | 4/5 |

**Figure 1** Representative Northern blots from samples of RNA isolates from four colorectal cancers and the corresponding normal colonic mucosa from the same surgical specimen. Hybridisation was performed with the p15E oligonucleotide probe described in the text. Lanes 1, 3, 5 and 7 are samples isolated from colorectal cancer. Lanes 2, 4, 6 and 8 are samples removed from proximal normal mucosa obtained from the paired surgical specimen, i.e. 1 & 2 were obtained from the same surgical specimen, as were 3 & 4, 5 & 6 and 7 & 8. Lanes 1, 5 and 7 were regarded as strongly positive. Lane 3 was regarded as weakly positive but this degree of positivity was typical in that observed for those five samples of normal colonic mucosa which were regarded as being positive. All other carcinoma specimens exhibited the same level of positive detection as seen in lanes 1, 5 and 7. All specimens designated as being negative for p15E mRNA gave blots identical with those in lanes 2, 4, 6 and 8.

ficance until the concentrations of the peptides were $10^{-6}$ M in culture ($P<0.01$, Student's $t$-test) (Figure 2). The concentration of these two peptides required to induce statistically significant ($P<0.01$) suppression of the proliferative response to recombinant IL-2 was also $10^{-6}$ M (Figure 3). In contrast, both peptides consistently ($P<0.01$) inhibited anti-CD3-induced lymphocyte proliferation at concentrations of $10^{-9}$ M or more (Figure 4).

### *Effects of FG-10 and FG-17 on polyclonal B-cell activation*

Neither of the two synthetic peptides cause any detectable inhibition of immunoglobulin production in response to polyclonal activation with pokeweed mitogen. Indeed, at a concentration of $10^{-6}$ M peptides the amount of immunoglobulin present in the supernatants of these polyclonally activated B-cells was slightly but significantly elevated ($P<0.05$, Figure 5).

### *Effects of FG-10 and FG-17 on the generation of lymphokine-activated killer (LAK) cells*

As can be seen from the data shown in Figure 6, under the conditions utilised in these experiments no inhibition of either LAK cell generation or the lytic efficacy of unmodified LAK cells was observed.

**Figure 2** Effects of different concentrations of the two synthetic peptides of p15E (FG10 ▇ and FG17 ▨) on human lymphocyte proliferation in the two-way mixed lymphocyte reaction. Vertical bars denote mean ± standard error of $^3$H-Thymidine uptake per well ($n=5$), after 140 h of culture (*$P<0.05$).

**Figure 3** Effects of different concentrations of the two synthetic peptides of p15E (FG10 ▇ and FG17 ▨) on human lymphocyte proliferation induced by recombinant Interleukin-2. Vertical bars denote mean ± standard error of $^3$H-Thymidine uptake per well ($n=5$), after 96 h of culture (*$P<0.05$).

### *Effects of FG-10 and FG-17 on the neutrophil oxidative burst*

Both peptides at a concentration of $10^{-6}$ M significantly suppressed the basal, zymosan-activated and PMA-activated neutrophil oxidative burst ($P<0.05$, Student's $t$-test (Figure 7)).

**Figure 4** Effects of different concentrations of the two synthetic peptides of p15E (FG10 ▇ and FG17 ▨) on human lymphocyte proliferation induced by anti-CD3 monoclonal antibody. Vertical bars denote mean ± standard error of $^3$H-Thymidine uptake per well ($n=5$), after 96 h of culture (*$P<0.05$).

**Figure 5** Effects of different concentrations of the two synthetic peptides of p15E (FG10 ▇ and FG17 ▨) on polyclonal B-cell activation induced by pokeweed mitogen. Vertical bars denote mean ± standard error of Ig content of supernatants from PWM-activated human peripheral blood mononuclear cells (*$P<0.05$).

**Figure 6** Effects of $10^{-6}$ M FG10 and FG17 on the induction of LAK cytotoxicity in three separate experiments. Data are expressed as AUC (area under the curve) units for each experiment. The data are linked for each individual experiment.

**Figure 7** Effects of control ▧, $10^{-6}$ M FG10 ■, and FG17 ▨ on basal, zymosan-induced and PMA-induced neutrophil oxidative burst. Vertical bars denote mean ± standard error of mean channel fluorescence obtained for the three separate experiments (* $P < 0.05$).

### Discussion

Our experiments have revealed that the majority of human colorectal and some gastric cancers contain cells which express RNA for a highly conserved retroviral sequence which is known to possess immunosuppressive properties (Cianciolo *et al.*, 1985; Harrell *et al.*, 1986; Ogasawara *et al.*, 1988). The size range of this sequence is consistent with that reported for other endogenous retroviral products (Repaske *et al.*, 1985). In the colon the majority of the macroscopically normal samples of mucosa failed to express p15E-relative mRNA. Of the five samples of non-malignant colorectal mucosa which were positive for p15E-related expression, four were histologically abnormal, two being afflicted with inflammatory bowel disease and two contained non-familial multiple polyps. A similar pattern was obtained for the small number of tissues obtained from the gastric cancer resectional specimens with all the tumours being positive for p15E-related mRNA, the only macroscopically normal positive mucosa being the seat of a severe chronic active gastritis with metaplasia.

The observations invite questions concerning the origin of the p15E-related mRNA seen in these tumours. Certainly p15E-related protein has been described in certain human tumour cells and rodent tumour cell lines (Cianciolo *et al.*, 1983; 1984). In addition sera from patients with certain haematopoietic malignancies contain a 74-kDa glycoprotein which contains a p15E-related epitope again suggesting that certain tumours of non-viral origin do express p15E-like material (Jacquemin & Strijckmans, 1985). The possibility that this material is expressed early during tumorigenesis might explain the fact that mRNA for this protein was observed in five out of six samples of non-malignant gastrointestinal mucosa, these five being histologically afflicted by conditions known to be pre-malignant *viz.* inflammatory bowel disease, colonic polyps, and gastritis with metaplasia. On the other hand, it could be argued that the positive isolates from the 'non-malignant' mucosae were due to contamination by tumour cells in all but one instance (the colon affected by Crohn's disease rather than malignant disease), since both the malignant and non-malignant samples were obtained from the same surgical specimens. However, we endeavoured to minimise this possibility by removing the 'normal' intestinal mucosa at a site at least 10 cm from a proximal site, away from the faecal stream.

An equally likely source for the p15E-like mRNA in these tissue samples is of course the inflammatory cells which they contain. It has been previously reported that murine monoclonal antibodies raised against an identical (but BSA-conjugated), peptide to FG-17 (CKS-17), have identified such material in inflamed lymphoid tissue (Tas *et al.*, 1991) and in malignant and chronic inflammatory conditions of the naso-oro-pharynx (Tan *et al.*, 1987; Scheeren *et al.*, 1992). The latter study by Scheeren *et al.* (1992) is of particular interest in that these authors were also unable to identify p15E-like material in normal intestinal mucosa using the 4F5 and 19F8 monoclonal antibodies described by Cianciolo *et al.* (1983). Antibodies to the CD4 epitope on human lymphocytes also bind to CKS-17 (Rothman *et al.*, 1990), but as yet there is no data which suggest that the antibodies to CKS-17 can bind to the CD4 molecule (Table I). However, only a small proportion of the inflammatory cell infiltrate in colorectal cancer is known to be CD4-positive, the majority being CD14-positive monocytes (Allen & Hogg, 1987). However, this latter observation presents us with further alternatives for CKS-17 antibody binding partners.

A 10-amino acid sequence from interferon-α (INF-α) has been identified which has a high level of sequence homology with CKS-17 (Table I) (Ruegg & Strand, 1990). This appears to be the smallest biologically active sequence of INF-α and has similar immunosuppressive properties to those which we describe here for our own synthetic peptides. CD14-positive monocytes are a potent source of INF-α. Using a computerised homology search we have found that there is a sequence identity between our 10 amino acid peptide and a sequence in tumour necrosis factor-α (TNF-α) (Table I). Again monocytes are a major cell of origin of TNF-α which has also been found to be present in colorectal cancer (Beissert *et al.*, 1989). However, these alternative proteins which may cross-react with CKS-17 specific antibodies have sufficiently different mRNA nucleotide sequences from p15E that they are very unlikely to hybridise with our oligonucleotide probe under the hybridisation conditions employed.

Although we demonstrated the presence of p15E-related mRNA at the site of colorectal and gastric cancers and in some inflamed and pre-malignant sites in these organs, the role of the proteins encoded by this endogenous message in humans is unknown. To investigate this we therefore constructed two synthetic peptides, FG-10 and FG-17 (Table I) which correspond to the endogenous p15E nucleotide sequence. At a concentration of $10^{-6}$ M both these peptides inhibited lymphocyte proliferation in mixed lymphocyte culture and in response to IL-2. Since the immunosuppressive properties of FG-10 and FG-17 did not differ, the active component must residue within the shorter ten amino acid sequence. By means of peptide screening of uncoupled peptides, Oostendorp *et al.* (1992) have recently described the antilymphoproliferative properties of a LDLLFL sequence which is fully contained in our FG-17 peptide but is only partially represented in the FG-10 peptide. Suppression of IL-2-induced proliferation occurred at identical concentrations in culture to those seen with our own peptides. However, in our studies the inhibition of anti-CD3-induced T-cell proliferation occurred at lower concentrations than those required to inhibit the MLR or IL-2 induced lymphocyte proliferation, suggesting that these peptides may specifically suppress T-cell function through the CD3-T cell receptor complex.

These peptides also inhibit the neutrophil oxidative burst. The fact that intracellular signalling for PMA-stimulated oxidative burst is mediated via protein phosphokinase C whereas that for T-cell proliferation is via the phosphatidylinositol 4,5-biphosphate hydrolysis pathway suggests that these peptides possess multiple mechanisms of action. Like the fusion proteins of HIV-1, these peptides may readily insert into the lipid bilayer of the cell membrane and interfere with a variety of ligand-induced signals. In contrast the lack of activity of these peptides on LAK cell induction or PWM-induced polyclonal immunoglobulin production suggests that their mode of action may be more specific than appears at first sight. It is also intriguing that our peptides exert their immunosuppressive activities without having to be conjugated to a carrier protein as was necessary for CKS-17 (Harrell *et al.*, 1986; Ogasawara *et al.*, 1988; Nelson *et al.*, 1989; Nelson & Nelson, 1990). As yet we are unable to explain this discrepancy.

Repaske *et al.* (1985) have described a full length endogenous human retroviral DNA which includes the code for a ten amino acid sequence (Table I) simliar to the FG-10 sequence. Whether or not this sequence codes for a peptide which possesses the same properties as FG-10 remains to be investigated and raises the question of the function of such sequences within the human genome. It is presumed that endogenous p15E-like proteins serve a useful purpose in regulating the cellular immune response. In the murine system p15E has been shown to be an important component of the inhibitory feedback circuit whose expression is induced not only by immune stimuli but also by glucocorticoids (Krieg *et al.*, 1989; Helmberg *et al.*, 1990). Since these sequences are highly conserved amongst the murine, feline and human species it is not unreasonable to suppose that these events are common to all the species.

We have made the assumption that the amino acid sequence constituting FG-10 is a functional component of a protein with the same functional attributes which are not lost during tertiary processing. Thus it might be argued that if the specific mRNA is of leukocyte origin, these data do provide evidence of the activation of an immune response against the tumour within the primary site, the presence of p15E-related mRNA representing the negative feedback regulatory arc of such a response. We are currently attempting to identify the presence of p15E-related mRNA in *in vivo* activated human lymph nodes in an attempt to provide evidence for or against this hypothesis. Conversely, the interaction of tumour cells with the infiltrating inflammatory cells may cause the tumour cells to produce immunoregulatory proteins, some of which may provide a survival advantage for the tumour cells. The outcome of this teleölogical argument must await the isolation of the protein that is translated by the mRNA that our oligonucleotide probes have identified and also immunohistochemical studies of the expression and distribution of such proteins using monoclonal antibodies which we are currently developing for this purpose. However, as has been suggested by others (Nelson *et al.*, 1985; Lindvall *et al.*, 1991), such reagents may also become important therapeutic tools as adjuncts to tumour immunotherapy in addition to providing scientific explanations for the failure of immunotherapy in so many instances.

### References

ALLEN, C. & HOGG, N. (1987). Elevation of infiltrating mononuclear phagocytes in human colorectal tumors. *JNCI.*, **78**, 465–470.

BASS, D.A., OLBRANTZ, P., SZEJDA, P., SEEDS, M.C. & McCALL, C.E. (1986). Subpopulations of neutrophils with increased oxidative product formation in blood of patients with infection. *J. Immunol.*, **136**, 860–866.

BEISSERT, S., BERGHOLZ, M., WAASE, I., LEPSIEN, G., SCHAUER, A., PFIZENMAIER, K. & KRONKE, M. (1989). Regulation of tumor necrosis factor gene expression in colorectal adenocarcinoma: *in vivo* analysis by *in situ* hybridization. *Proc. Natl Acad. Sci. USA*, **86**, 5064–5068.

CIANCIOLO, G.J., COPELAND, T.D., OROSZLAN, S. & SNYDERMAN, R. (1985). Inhibition of lymphocyte proliferation by a synthetic peptide homolgous to retroviral envelope protein. *Science*, **230**, 453–455.

CIANCIOLO, G.J., LOSTROM, M.E., TAM, M. & SNYDERMAN, R. (1983). Murine malignant cells synthesize a 19,000 dalton protein which is physicochemically and antigenically related to the immunosuppressive retroviral protein P15E. *J. Exp. Med.*, **158**, 885–890.

CIANCIOLO, G.J., PHIPPS, D. & SNYDERMAN, R. (1984). Human malignant and mitogen-transformed cells contain retroviral P15E-related antigen. *J. Exp. Med.*, **159**, 964–969.

DYE, J.F., SOMERS, S.S. & GUILLOU, P.J. (1991). Simplified quantitation of cytotoxicity by integration of specific lysis against effector cell concentration at a constant target cell concentration and measuring the area under the curve. *J. Immunol. Meth.*, **138**, 1–13.

EBERT, E.C., ROBERTS, A.I., O'CONNELL, S.M., ROBERTSON, F.M. & NAGASE, H. (1987). Characterisation of an immunosuppressive factor derived from colon cancer cells. *J. Immunol.*, **138**, 2161–2168.

GUILLOU, P.J. (1989). The immunological status of the cancer patients. *Curr. Opin. Immunol.*, **1**, 913–916.

GUILLOU, P.J. (1991). Immunotherapy for cancer. *Br. J. Surg.*, **78**, 1281–1282.

GUILLOU, P.J., SEDMAN, P.C. & RAMSDEN, C.W. (1989*a*). Inhibition of lymphokine-activated killer cell generation by cultured tumor cell lines *in vitro*. *Cancer Immunol. Immunother.*, **28**, 43–53.

GUILLOU, P.J., RAMSDEN, C.W., SOMERS, S.S. & SEDMAN, P.C. (1989*b*). Suppression of the generation of lymphokine-activated killer (LAK) cells by serum-free supernatants of *in vitro* maintained tumor cell lines. *Br. J. Cancer*, **59**, 515–521.

HARRELL, R.A., CIANCIOLO, G.J., COPELAND, T.D., OROSZLAN, S. & SNYDERMAN, R. (1986). Suppression of the respiratory bursts of human monocytes by a synthetic peptide homologous to envelope proteins of human and animal retroviruses. *J. Immunol.*, **136**, 3517–3520.

HELMBERG, A., FASSLER, R., GELEY, S., JOHRER, K., KROEMER, G., BOCK, G. & KOFLER, R. (1990). Glucocorticoid-regulated gene expression in the immune system. Analysis of glucocorticoid-regulated transcripts from the mouse macrophage-like cell line P388D1. *J. Immunol.*, **145**, 4332–4337.

JACQUEMIN, P.C. & STRIJCKMANS, P. (1985). Detection of a retrovirus-related glycoprotein in immune complexes from patients with hematopoietic disorders. *Int. J. Cancer*, **36**, 535–539.

KRIEG, A.M., GAUSE, W.C., GOURLEY, M.F. & STEINBERG, A.D. (1989). A role for endogenous retroviral sequences in the regulation of lymphocyte activation. *J. Immunol.*, **143**, 2448–2451.

LEONG, S.P., MULLER, J., YETTER, R.A., GORELIK, E., TAKAMI, T. & HEARING, V.J. (1988). Expression and modulation of a retrovirus-associated antigen by murine melanoma cells. *Cancer Res.*, **48**, 4954–4958.

LINDVALL, M. & SJOGREN, H.O. (1991). Inhibition of rat yolk sac tumour growth *in vivo* by a monoclonal antibody to the retroviral molecule p15E. *Cancer Immunol. Immunother.*, **33**, 21–27.

MONSON, J.R.T., RAMSDEN, C.W., GILES, G.R., BRENNAN, T.G. & GUILLOU, P.J. (1987). Lymphokine-activated killer cells in patients with gastrointestinal cancer. *Gut*, **28**, 1420–1425.

MONSON, J.R.T., RAMSDEN, C.W. & GUILLOU, P.J. (1986). Decreased interleukin-2 production in patients with gastrointestinal cancer. *Br. J. Surg.*, **73**, 483–486.

NELSON, M. & NELSON, D. (1990). Inhibition of interleukin-2 production by tumor cell products and by CKS-17, a synthetic retroviral envelope peptide. *Cancer Immunol. Immunother.*, **30**, 331–341.

NELSON, M., NELSON, D.S., CIANCIOLO, G.J. & SNYDERMAN, R. (1989). Effect of CKS-17, a synthetic retroviral envelope peptide on cell-mediated immunity *in vivo*: immunosuppression, immunogenicity and relation to immunosuppressive tumor products. *Cancer Immunol. Immunother.*, **30**, 113–118.

NELSON, M., NELSON, D.S., SPRADBROW, P.B., KUCHROO, V.K., JENNINGS, P.A., CIANCIOLO, G.J. & SNYDERMAN, R. (1985). Successful tumour immunotherapy: possible role of antibodies to anti-inflammatory factors produced by neoplasms. *Clin. Exp. Immunol.*, **61**, 109–117.

OGASAWARA, M., CIANCIOLO, G.J., SNYDERMAN, R., MATANI, M., GOOD, R.A. & DAY, N.K. (1988). Human INF-$\gamma$ production is inhibited by a synthetic peptide homologous to retroviral envelope protein. *J. Immunol.*, **141**, 614–619.

OOSTENDORP, R.A.J., SCHAAPER, W.M.M., POST, J. VON BLOMBERG, B.M.E., MELOEN, R.H. & SCHEPER, R.J. (1992). Suppression of lymphocyte proliferation by a retroviral p15E-derived hexapeptide. *Eur. J. Immunol.*, **22**, 1505–1511.

REPASKE, R., STEELE, P.E., O'NEILL, R., RABSON, A.B. & MARTIN, M.A. (1985). Nucleotide sequence of a full-length human endogenous retroviral segment. *J. Virol.*, **54**, 764–772.

ROTHMANN, J., HASSAN, N.F., CAMPBELL, D.E., KAMANI, N. & DOUGLAS, S.D. (1990). Synthetic peptide homologous to the envelope protein of retroviruses share a cross-reacting epitope with the CD4 receptor. *J. Clin. Microbiol.*, **28**, 112–115.

ROSENBERG, S.A., LOTZE, M.T., YANG, J.C., AEBERSOLD, P.M., LINEHAN, W.M., SEIPP, C.A. & WHITE, D.E. (1989). Experience with the use of high dose interleukin-2 in the treatment of 652 cancer patients. *Ann. Surg.*, **210**, 474–485.

RUEGG, C.L. & STRAND, M. (1990). Identification of a decapeptide region of human interferon-α with antiproliferative activity and homology to an immunosuppressive sequence of the retroviral transmembrane protein P15E. *J. Interferon Res.*, **10**, 621–626.

SCHEEREN, R.A., OOSTENDORP, R.A., VAN DER BAAN, S., KEEHNEN, R.M., SCHEPER, R.J. & MEIJER, C.J. (1992). Distribution of retroviral p15 related proteins and non-neoplastic human tissues, and their role in the regulation of the immune response. *Clin. Exp. Immunol.*, **89**, 94–99.

SNYDERMAN, R. & CIANCIOLO, G.J. (1984). Immunosuppressive activity of the retroviral envelope protein and its possible relationship to neoplasia. *Immunol. Today*, **5**, 240–244.

SOMERS, S.S., DYE, J.F. & GUILLOU, P.J. (1991). Comparison of transforming growth factor-beta and a human tumour-derived suppressor factor. *Cancer Immunol. Immunother.*, **33**, 217–222.

SPRANG, S.R. & ECK, M.J. (1992). The 3-D structure of TNF. In *Tumor Necrosis Factors – the Molecules and their Emerging Role in Medicine*. Buetler, B. (ed.). Raven Press: New York. pp. 11–32.

STEWART, M.A., WARNOCK, M., WHEELER, A., WILKIE, N., MULLINS, J.I., ONIONS, D.A. & NEILL, J.C. (1986). Nucleotide sequences of a feline leukemia virus subgroup A envelope gene and long terminal repeat and evidence for the recombinational origin of subgroup B viruses. *J. Virol.*, **58**, 825–834.

TAS, M., DE HAAN-MEULMAN, M., KABEL, P.J. & DREXHAGE, H.A. (1991). Defects in monocyte polarization and dendritic cell clustering in patients with Graves' disease: a putative role for a non-specific immunoregulatory factor related to retroviral P15E. *Clin. Endocrinol.*, **34**, 441–448.

TAN, I.B., DREXHAGE, H.A., MULLINK, R., HENSEN-LONGMANS, S., DE HAAN-MEULMAN, M., SNOW, G.B. & BALM, A.J.M. (1987). Immunohistochemical detection of retroviral P15E-related material in carcinomas of the head and neck. *Otolaryngol. Head Neck Surg.*, **96**, 251–255.

WAKEFIELD, C.H., FOULDS, S., CAREY, P.D., MONSON, J.R.T. & GUILLOU, P.J. (1993). Polymorphonuclear leukocyte activation: an early marker of the post-surgical sepsis response. *Arch. Surg.*, **128**, 217–222.

WEST, W.H., TAUER, K.W., YANELLI, J.R., MARSHALL, G.D., ORR, D.W., THOMAN, G.B. & OLDHAM, R.K. (1987). Constant infusion recombinant interleukin-2 in adoptive immunotherapy of advanced cancer. *N. Engl. J. Med.*, **316**, 898–905.